# Supplementary material for: Smart Wearable Systems for the Remote Monitoring of Selected Vascular Disorders of the Lower Extremity: A Systematic Review
Source: Int J Environ Res Public Health. 2022 Nov 18;19(22):15231. doi: 10.3390/ijerph192215231 (PMC9690814; doi:10.3390/ijerph192215231)
Supplement: Supplementary file 1 [file ijerph-19-15231-s001.zip › ijerph-1956437-supplementary.pdf]

Supplementary Table S1. Overall description of the included studies.

Author, Year, Country	*Disease*	*Objective*	*Number of Users Tested*	*Mean Age*	*Study Period*	*Outputs of the Study*
Armstrong et al. (2004), United States	Diabetic foot ulcer	To evaluate the role of activity in the development of neuropathic foot ulceration in diabetic patients.	100 diabetic individuals	68.5	25 weeks. Patients were instructed to wear the device at all times during the day and night.	Individuals with diabetes who develop ulceration may actually have a lower overall activity than those with no ulceration. Intelligent activity monitors designed to identify deleterious variations in activity and to notify patients and providers might prove to reduce risk for ulceration in very-high-risk population.
Chidean et al. (2018), Spain	Intermittent claudication	To use a full band spectral analysis (up to 15 Hz) and the fundamental frequency in order to completely characterize gait for both PAD and control patients	8 PAD (Peripheral arterial disease) patients and 10 controls.	62.3	Participants had to walk, free-fashion for 10 minutes, apart from participants who had to stop walking due to their condition. In these cases, experiment lasted as long as possible.	Full spectral analysis allowed to better characterize gait in PAD patients than classical spectral analysis and allowed to better discriminate between PAD and control patients, also showing promising results to assess severity of PAD.
Clarke et al. (2013), United Kingdom	Intermittent claudication	To develop a method of event-based analysis that quantifies the fragmented nature of walking bouts in individuals with intermittent claudication [IC] and compare outcomes with age and gender-matched healthy controls	Individuals with IC (n = 30) and controls matched for age and gender (n = 30).	67.2	7-day continuous ambulatory monitoring.	7-day continuous ambulatory monitoring is able to objectively quantify the fragmented nature of walking bouts in patients with IC. Of particular importance is the identification of the classic fragmented stop/start walking pattern universally described by IC patients.
Crews et al. (2012), United States	Diabetic foot ulcer	To compare the offloading capacity of walkers with varying height (knee, ankle, and shoe levels).	11 diabetic subjects with moderate to high risk of ulceration.	51.4	Subjects completed four 20 m walking trials. Subjects performed one trial with each walker and one trial with an athletic shoe.	The ankle-high RCW may prove to provide better outcomes in treating DFU. It may also be a viable choice for individuals with significant edema at the proximal end of the leg.
De León Rodriguez et al. (2013), Switzerland	Diabetic foot ulcer	To evaluate the impact of a new walking strategy learned by biofeedback on plantar pressure distribution under both feet in patients with diabetic peripheral neuropathy	A total of 25 subjects with diabetes mellitus.	57.8	10 days	Terminally augmented visual biofeedback helps reduce plantar pressure in diabetic patients with peripheral neuropathy without foot deformity to a safe level and without increasing the pressure at any other areas of both feet.
Du et al. (2021), China	Diabetic foot ulcer	To use wearable sensors in order to explore changes in gait and balance parameters, over time, in elderly	6 patients with type 2 diabetes who recently	55–80	Not specified.	The wearable sensor-based monitoring was convenient, feasible, and effective, providing an empirical quantitative assessment of gait and

		patients at high risk of diabetic foot, wearing different types of footwear.	recovered from DFU			balance changes in elderly diabetic patients before and after wearing offloading footwear. Gait and balance ability worsens over time in elderly patients with diabetic foot.
Fokkenrood et al. (2014), The Netherlands	Intermittent claudication	To validate daily activities using a novel type of tri-axial accelerometer (Dynaport MoveMonitor) in patients with IC	27 IC patients	67	Not specified.	A tri-axial Dynaport activity monitor provides accurate information on a diverse set of daily activities in patients with IC when compared with a video technique.
Gordon et al. (2020), United States	Diabetic foot ulcer	To assess the accuracy of once-daily foot temperature monitoring for predicting foot ulceration in diabetic patients with recent wounds and partial foot amputation.	129 participants	61.8	34 weeks	Once-daily foot temperature monitoring is clinically effective for the early detection of DFUs for patients who are at high risk for diabetic foot complications, those with recent wounds and partial foot amputations.
Henricson et al. (2021), Sweden	Venous ulcer	To evaluate the ability of a newly developed moisture sensor to detect moisture in relation to the absorbing capacity of the dressing.	5 patients	75	Three dressing changes.	The combination of dressing and moisture sensor is promising. A scientific evaluation of potential home monitoring of wounds by a moisture sensor is recommended.
Kekonen et al. (2021), Finland	Venous ulcer	To examine the feasibility of the bioimpedance measurement-based method and the measurement system in monitoring healing of hard-to-heal wounds.	6 patients with venous ulcers	65	Healing of ulcers was monitored until the complete re-epithelialization. Follow-up ranged between19 to 106 days.	The bioimpedance measurement-based method is a promising quantitative tool for monitoring the status of venous ulcers.
Kekonen et al. (2019), Finland	Venous ulcer	To present a prototype of a multi-electrode dressing and the successful results of long-term monitoring of an acute wound healing from beneath the primary dressings.	1 patient	Not informed.	120 hours	The initial decrease of the skin impedance is most likely due to the skin absorbing moisture delivered by the hydrogel pads. It is well-capable for long term monitoring. In the future hydrogels should be incorporated into the dressing in the production phase.
Kelechi et al. (2020), United States	Venous ulcer	To investigate the use of an mHealth application, self-management physical activity intervention FOOTFIT, with an added patient-provider communication feature (FOOTFIT +) designed to strengthen the lower extremities of minimally ambulatory individuals with venous leg ulcers.	24 patients	60.7 (FOOTFIT) + 69.1 (FOOTFIT+)	6 weeks	The greatest improvement was noted in the FOOTFIT group for dorsiflexion of the right ankle, whereas strength decreased in both ankles for dorsiflexion and plantar flexion in the FOOTFIT +.
Lauret et al. (2014), The Netherlands	Intermittent claudication	To quantify daily physical activity level and energy expenditure of newly	94 IC patients and 36 healthy individuals	69	1 week	Future studies should focus on the effects of different treatment strategies on PA.

		diagnosed patients with IC and healthy controls.				
Madavi et al. (2016), India	Diabetic foot ulcer	To design and implement a wearable device for diabetic foot patients to detect the level of foot ulcers-related issues along with daily exercise level monitoring.	unknown	Not informed.	unknown	The proposed system can be used for rehabilitation, being able to improve gait. Athletic people also use this for guidance of pressure on foot.
Mancilla et al. (2021), Mexico	Diabetic foot ulcer	To retrieve temperature differences between the sole and regions with abnormal temperature patterns, based on infra-red thermography and image processing.	12 patients	Not informed.	unknown	The combination of automatic temperature difference estimation and segmentation could be a scalable method for other illness diagnostics requiring quantitative support. Also, the automatic processing would allow the study of large numbers of samples and patients without the need for human intervention, which could save time for the physiologist. The evolution of this work is intended to include statistical studies to support the hypothesis of prevention of DF in terms of the temperature difference and thermal patterns.
Mori et al. (2012), Japan	Diabetic foot ulcer	To establish a simultaneous in-shoe measurement system of plantar pressure and shear force during a gait.	3 patients, 5 adult subjects, 50 diabetic patients	67.4	Tests performed individually, with no follow-up.	Results have suggested that reducing stress in this region is necessary in the care of diabetic patients.
Owings et al. (2009), United States	Diabetic foot ulcer	To measure in-shoe plantar pressures and other characteristics in a group of neuropathic patients with diabetes who had prior foot ulcers which had remained healed.	49 patients with prior plantar foot ulcer	62.9	An experimental session consisting of a foot examination, interviews regarding activity level and compliance and measurement of plantar pressures during barefoot walking and within the patients' most commonly worn shoes (typically prescription footwear).	Authors propose that the mean value for in-shoe pressures reported in these patients be used as a target in footwear prescription for patients with prior ulcers. Although plantar pressure is only one factor in a multifaceted strategy to prevent ulcer recurrence, the quantitative focus on pressure reduction in footwear is likely to have beneficial effects.
Schneider et al. (2019), United States	Diabetic foot ulcer	To examine the feasibility of a low-intensity, technology-based behavioral intervention to increase activity in adults at risk for DFUs.	12 participants at risk for a DFU (adult over 21, with diabetes).	59.9	Participants received four in-person exercise and behavioral counseling sessions over 2-3 weeks, supplemented with use of an activity monitor (to track steps) and text	Individuals at risk for a DFU might benefit from a minimally intensive, technology-based intervention to increase their physical activity. Future research comparing the intervention to usual care is warranted.

					messages (to reinforce behavioral strategies) for an added 8 weeks.	
Sieminski et al. (1997), United States	Intermittent claudication	To assess the magnitude of the reduction in free-living daily physical activity of claudicants compared with age-matched controls, and to examine the relationship between the severity of peripheral arterial occlusive disease (PAOD) and free-living daily physical activity.	85 PAOD and 59 non-PAOD subjects	67.3	2 week days	Results from this study confirm the relationship between smoking status and physical activity value obtained from the accelerometer and indicate that age and weight also are important covariates of activity. Future studies are needed to identify other influential factors of physical activity in the community setting.
Torreblanca González et al. (2021), Spain	Diabetic foot ulcer	To develop a system, a smart sock, capable of measuring temperature at various points on the foot, to record these measurements during gait by using a smartphone and finally to analyze the data and alert the patient where necessary	93 subjects	Not informed.	Variable	This smart sock is able to continuously obtain temperatures at several points of the foot, this leaves open the possibility of advancing the study of this disease in the near future.
Watanabe et al. (2017), Japan	Diabetic foot ulcer	To evaluate the forefoot load (FL) in daily walks of diabetic patients using a wearable motion sensor attached to each foot, so as to compare the FL between daily life environment and laboratory setting	10 healthy subjects + 2 subjects with diabetes	40.5	Approximately 3 hours	Daily walk measurement using wearable motion sensors appears feasible and safely induces no adverse events in patients with diabetes. The algorithm was able to estimate the forefoot load during walking on a level corridor with more than moderate accuracy. However, the estimation accuracy was not consistent among the subjects in stair walking and slope walking.
Wrobel et al. (2014), United States	Diabetic foot ulcer	To evaluate the effect of a novel shear-reducing insole on the thermal response to walking, balance, and gait.	Twenty-seven subjects with diabetes (type 1 or type 2)	65.1	Variable	The authors found significant reductions in forefoot and midfoot temperature increases after known walking stress using a novel shear-reducing insole when compared to standard insoles. Future footwear studies should also consider measuring thermal and sudomotor function changes.
Zaffar et al. (2016), Pakistan	Diabetic foot ulcer	To present the electronic design of a low-cost, non-invasive, patient centric device, for early diagnosis of Diabetic Peripheral Neuropathy (DPN).	8 diabetic patients	Not informed.	Variable	The companion application provides a graphical display of data for the patients which is also uploaded to the cloud for online access to the physicians. A medical study for detailed clinical results is currently underway and will be presented in near future. The device has the potential to directly improve the life standard of millions of diabetic patients worldwide.

Ata et al. (2018), United States	Intermittent claudication	To assess the feasibility of our 6-min walk test (6MWT) app, “VascTrac,” to serve as a platform for performing 6MWTs in patients with PAD by (1) evaluating the accuracy of the iPhone’s step and distance tracking algorithms in the PAD population, and (2) assessing the concordance of the iPhone algorithms with the ActiGraph GT9X.	One hundred and fourteen individuals	69.5	6-min walk test	Authors demonstrated that the pedometer present in a commercial mobile phone provided a distance estimation algorithm with poor accuracy in patients with PAD, likely due to lack of correction for an individual’s stride length. However, its step counting algorithm is highly concordant with the reference standard.
Banks et al. (2020), United States	Diabetic foot ulcer	To present a case series supporting the use of remote temperature monitoring for early identification of DFUs	4	76	4-month period	Results of this case series support the use of telemedicine RTM by a smart thermometric foot mat for the early identification of inflammation to prompt clinical evaluation and intervention. More research is needed to further quantify the benefits of the RTM mat for both prevention and early identification of DFUs and other inflammatory diabetic foot complications.
Begg et al. (2012), Australia	Diabetic foot ulcer	The aim of this proof-of-concept study was to determine the feasibility of a new method to directly measure the load between the cast wall and the lower leg interface using capacitance sensors.	2	26	The participants walked at a walking speed of approximately 0.4 ± 0.04 m/sec over a 9 m walkway	The direct measurement approach demonstrated in this study showed that it is possible to measure the load between the cast wall and the lower leg. However, the methodology requires repeating in a larger sample of participants with plantar foot ulceration.
Bus et al. (2012), The Netherlands	Diabetic foot ulcer	To assess the validity and feasibility of a new temperature-based adherence monitor to measure footwear use.	11 healthy subjects + 14 diabetic patients	56.2	7-days	The study showed that valid data can be acquired when using the monitor to assess footwear use in healthy subjects, and that together with step activity monitoring, its use is feasible in neuropathic diabetic subjects.
Chatwin et al. (2018), United Kingdom	Diabetic foot ulcer	Case report describing the use of an innovative plantar pressure feedback system, allowing the description of its effect on plantar pressures in both feet. we captured the effects of a foreign object	1	59	Both pre- and post-screw time periods represent 10 days of data collection before and immediately after the screw was embedded.	This case study provides an interesting insight into biomechanical alterations due to a foreign object in the shoe of a diabetes participant with peripheral neuropathy and ankle fusion. The unknown presence of the screw resulted in significant increases in plantar pressure to the contralateral foot, thus increasing its risk of ulceration.
Chaudru et al. (2019), France	Intermittent claudication	To develop, assess the feasibility of, and determine the clinical validity of an event-based analysis method using wearable monitors to quantify	23	60	7 days	The proposed methodology monitor could potentially facilitate the discrimination of marker events (WPMs and SIWPs). Authors acknowledge

		walking pain manifestations (WPMs) and stops induced by walking pain (SIWPs) during daily life walking in people with peripheral artery disease (PAD).				that the methodology should be applied and tested in another population of PAD participants.
Cornelis et al. (2021), Belgium	Intermittent Claudication	To assess patient satisfaction and acceptability of a structured HBET program using wearable technology and elastic band resistance exercises.	20 patients with IC.	64.6	Participants were instructed to complete 3 walking sessions and 2 elastic band resistance exercise sessions per week in their home environment during a 4-week period.	This observational pilot study has shown that patients with IC are satisfied and accept technology to monitor and guide a home-based combined exercise program through remote feedback. Participants did not prefer resistance training over walking exercise; however, a general positivity toward the combined intervention was reflected in clinically relevant improvements in subjectively reported walking distances and quality of life.
Duscha et al. (2018), United States	Intermittent claudication	To determine the effects on functional capacity and physical activity patterns of a 12-week mHealth program in PAD patients with IC.	20	69.4	12 weeks	A 12-week mHealth program in PAD patients with IC can improve peak VO2 and claudication onset time; and mHealth interventions represent a promising alternative therapy for those patients who cannot participate in supervised exercise.
Frykberg et al. (2017), United States	Diabetic foot ulcer	To evaluate a novel remote foot-temperature monitoring system to characterize its accuracy for predicting impending diabetic foot ulcers (DFU) in a cohort of patients with diabetes with previously healed DFU	129	61.8	34 weeks. Participants were instructed to place the device in a convenient location within the home and to stand on it for 20 s daily. Participants returned devices to the enrolling site upon completion of or withdrawal from the study, at which time each participant completed a foot exam, a final scan with the study device, and a brief usability questionnaire.	Given the encouraging study results and the significant burden of DFU, use of this mat may result in significant reductions in morbidity, mortality, and resource utilization.
Gardner et al. (2010), United States	Intermittent claudication	To determine the effect of claudication pain on temporal and spatial gait characteristics, and on ambulatory symmetry at preferred and	28 patients with unilateral intermittent claudication	71	Variable	Claudication pain slows ambulatory velocity at preferred and rapid paces and increases asymmetry when ambulatory function is challenged with rapid walking. The reduced ambulatory speed with the

		rapid self-selected walking paces in patients with unilateral PAD.				development of claudication pain may be an adaptation to elicit a safer and less destabilizing gait pattern.
Gardner et al. (2007), United States	Intermittent claudication	To compare the patterns of ambulatory activity in subjects with and without intermittent claudication.	98 subjects limited by intermittent claudication and 129 controls	66 (IC group) + 64 (Control group)	7 days	Intermittent claudication is associated with lower total daily ambulatory activity owing both to less time ambulating and to fewer strides taken while ambulating, particularly at moderate and high cadences. Future research is needed to determine whether the pattern of daily ambulatory activity changes after interventions designed to improve claudication distances, such as exercise rehabilitation, medication therapy, and interventional procedures.
Gardner et al. (2008), United States	Intermittent claudication	To determine the association between daily ambulatory activity patterns and exercise performance in patients with intermittent claudication.	133	67	1 week	Daily ambulatory cadences are associated with severity of intermittent claudication, as measured by ACD and ICD, but not with peripheral hemodynamic measures.
Gardner et al. (2022), United States	Intermittent claudication	To determine whether patients with claudication who reported performing either light intensity physical activity (LPA) or moderate-to-vigorous intensity physical activity (MVPA) would have higher levels of objectively determined physical activity and better physical function, health-related quality of life (HRQoL), and vascular measures, consisting of exercise time to minimum calf muscle oxygen saturation (StO2) and high-sensitivity C-reactive protein, than patients who reported being physically sedentary.	269	65.5 (Sedentary group) + 65.2 (LPA group) + 67.1 (MVPA group)	1 week	Patients with claudication who reported performing light intensity physical activity (LPA) had greater amounts of objectively determined physical activity levels and better physical function, HRQoL, and vascular measures than those who reported being physically sedentary. The clinical significance is that our results have shown that engaging in any physical activity, even at relatively light intensity, is associated with favorable health and vascular measures for patients with claudication.
Gardner et al. (2014), United States	Intermittent claudication	To compare changes in primary outcome measures of claudication onset time (COT) and peak walking time (PWT), and secondary outcomes of submaximal exercise performance, daily ambulatory activity, vascular function, inflammation, and calf muscle hemoglobin oxygen saturation	180	65 (Attention-Control Group) + 65 (Supervised-Exercise Group) + 67 (Home-	12 weeks	NEXT Step home exercise utilizing minimal staff supervision has low attrition, high adherence, and is efficacious in improving COT and PWT, as well as secondary outcomes of submaximal exercise performance, daily ambulatory activity, vascular function, inflammation, and calf muscle StO2 in symptomatic patients with PAD.

		(StO2) in patients with symptomatic peripheral artery disease (PAD) following new exercise training using a step watch (NEXT Step) home-exercise program, a supervised exercise program, and an attention-control group.		Exercise Group)		
Gardner et al. (2011), United States	Intermittent claudication	To compare changes in exercise performance and daily ambulatory activity in peripheral artery disease patients with intermittent claudication after a home-based exercise program, a supervised exercise program, and usual-care control.	119	65 (Control Group) + 66 (Supervised-Exercise Group) + 65 (Home-Exercise Group)	12 weeks	A home-based exercise program, quantified with a step activity monitor, has high adherence and is efficacious in improving claudication measures similar to a standard supervised exercise program. Furthermore, home-based exercise appears more efficacious in increasing daily ambulatory activity in the community setting than supervised exercise.
Gernigon et al. (2014), France	Intermittent claudication	To determine the clinical applicability of a global positioning system (GPS)-monitored community-based walking ability assessment in a large cohort of patients with peripheral artery disease (PAD).	218	65	2 weeks	GPS is applicable for the nonsupervised multicenter recording of walking ability in the community. In the future, it may facilitate objective community-based assessment of walking ability, allow for the adequate monitoring of home-based walking programs, and for the study of new dimensions of walking in PAD patients with intermittent claudication.
Kelechi et al. (2020), United States	Venous ulcer	To establish the feasibility of a mobile health (mHealth) physical activity exercise app for individuals with VLUs to improve lower leg function	24	60.7 (FOOTFIT) + 69.1 (FOOTFIT+)	6 weeks	The findings of this study suggest that despite initial interest in using the app, several components of the program as originally designed had limited acceptability and feasibility. Future refinements should include the use of more modern technology including smaller wearable accelerometers, mobile phones or tablets with larger screens, an app designed with larger graphics, automated reporting for providers, and more engaging user features.
Killeen et al. (2018), United States	Diabetic foot ulcer	Case series to illustrate the use of a Remote temperature monitoring (RTM) foot mat for the early detection and prevention of DFUs in patients with a history of DFUs.	Three patients were chosen via retrospective chart analysis from the authors' high-risk podiatry clinic.	72	Patients were instructed to stand on the mat barefoot for 20 seconds at about the same time each day. The largest follow-up took 22 weeks (until asymmetry episodes were solved).	Results of this case series support the use of RTM by a smart thermometric foot mat for the early identification of inflammation to prompt clinical evaluation and intervention. Although this case series is limited in scope by its small sample size, the findings are consistent with literature suggesting the value of daily foot temperature monitoring for high-risk patients.

Lin et al. (2020), Taiwan	Diabetic foot ulcer	To use wearable near-infrared spectroscopy (NIRS) to determine the effect of Buerger exercises on diabetic foot ulcer (DFU) healing.	50	66.1	1 year	Although DFUs remain a challenge to treat, NIRS may prove valuable in predicting wound healing by identifying risk factors for poor wound prognosis, such as reduced hemoglobin and tissue blood volume after exercise.
Lott et al. (2005), United States	Diabetic foot ulcer	Case report to illustrate how sudden changes in weight-bearing activity may have affected ulcer recurrence in a patient with DM and how the physical stress theory (PST) relates to ulcer recurrence for this patient	One 66-year-old patient with a history of DM, peripheral neuropathy, and recurrent plantar ulcers.	66	To quantify the number of repetitions of stress exposure to the plantar surface of the foot from walking, the patient wore an activity monitor for 2 weeks during intervention with TCC. Approximately 3 weeks after wound healing, the patient returned per our request for plantar pressure testing and another activity level assessment.	Rapid change in activity level may have an effect on cumulative stress and the risk of ulcer recurrence.
Moulaei et al. (2021), Iran	Diabetic foot ulcer	To develop a smart wearable device to monitor these parameters to prevent diabetic foot.	5 (four patients with diabetes and one without diabetes)	> 46	Around 4 minutes	This device measure and monitor pressure in the first metatarsals head, the fifth metatarsals head, toe, and heel on each foot sole. Moisture and temperature were also measured in the mid foot by the sensors. Future studies should be conducted to evaluate the usability of the system and patients' satisfaction with wearable shoes for diabetic foot ulcer
Mueller et al. (1994), United States	Diabetic foot ulcer	To determine whether instructing a subject to walk using a hip strategy would reduce forefoot peak plantar pressures (PPP) and change the kinematics of walking during a single session of testing	7 subjects with diabetes mellitus and 6 age-matched controls	56.4	Variable	This study demonstrated that instructing subjects with diabetes mellitus and a recent history of forefoot ulceration to walk using a hip strategy can significantly reduce forefoot PPP during a single session
Nasr et al. (2002), United Kingdom	Intermittent claudication	To evaluate the role of pedometers in the assessment of patients with intermittent claudication by comparing them with treadmill testing, post-exercise ankle brachial pressure index (ABPI) and physical function questionnaires.	Fifty patients with at least 6 months history of stable intermittent claudication	67	Variable	In conclusion pedometer estimation of physical activity in the community may offer a more objective estimation of the degree of physical disability to the patient than either physical function questionnaires or treadmill testing alone, especially as they require less supervised time than treadmill testing and questionnaires and can be

						self-administered. For future clinical trials of claudication therapy, it has been suggested that laboratory assessments should serve only as secondary endpoints
Ning et al. (2021), United States	Venous ulcer	To investigate the changes in pressure over time under three different compression bandages and compare the temporal patterns of pressure changes among them.	10 healthy subjects and 20 outpatients with venous ulcers	70.6	7 days	The interface pressure decreased over time under all three studied bandages; monitoring the interface pressure, which would allow for adjusting or changing the bandage at an accurate time point, is essential to main-tain a desirable interface pressure during compression therapy.
Reyzelman et al. (2018), United States	Diabetic foot ulcer	to assess the accuracy of sensors used in daily wear socks, obtain user feedback on how comfortable sensor-embedded socks were for home use, and examine whether observed temperatures correlated with clinical observations.	35 patients with diabetes	62	3-24 hours	The socks can reliably and consistently collect temperature data from the wearer's feet, which are consistent with clinical observations. Future studies will be statistically powered to collect and analyze temperatures and correlate the findings to patient outcomes
Reyzelman et al. (2022), United States	Diabetic foot ulcer	To evaluate the temperature data for patients that presented with a diabetic foot injury while using a sock-based remote temperature monitoring device	5 participants with a diabetes-related lower extremity injury and 26 patients	66.0 (Intervention) + 70.4 (Control)	15 days	The results of this study suggest temperature monitoring using a sock form factor may be a predictor of a developing foot injury. Further study is needed with greater numbers of patients to establish the optimal early detection period.
Sarnow et al. (1994), United States	Diabetic foot ulcer	To measure in-shoe foot pressures in diabetic patients and healthy subjects and compare them with the foot pressures when they walked without their shoes.	44 type I or type II diabetic patients and 65 healthy subjects	55.5 (Diabetic group) + 50.9 (Control group)	Variable	The shoes of diabetic patients provided a higher-pressure reduction than did those of the control group, but the number of feet with abnormally high pressures did not change. The F-Scan system may be particularly helpful in designing footwear suitable for diabetic patients with at-risk feet
Scholten et al. (2022), United States	Diabetic foot ulcer	To evaluate utilization data for patients who were prescribed smart socks as remote temperature monitoring devices.	160 patients	69.9	7-month period	This study shows a high level of utilization and compliance for a smart sock remote temperature monitoring device. Further studies with larger patient groups and a longer follow-up period are warranted to better understand the sustained adherence to RPM among patients with diabetes.
Wang et al. (2021), Chine	Diabetic foot ulcer	To present a novel low-cost shoe system (a pressure-sensitive flexible sensor based wireless footwear system) for daily monitoring of plantar pressure in diabetics	15 subjects (5 in each of non-diabetic healthy controls (HC) group, dia-betic controls (DC)	48.5 (Healthy controls) + 55.8 (Diabetic controls) + 59 (Diabetic	Variable	Future work includes collecting more clinical plantar pressure data for diabetic patients, adopting more features and classification techniques for predictive classification, and conducting cross-subject classification using transfer learning.

			without peripheral neuropathy group and di–abetic with neuropathy (DN) group)	with Neuropathy)		
Yavuz et al. (2020), United States	Diabetic foot ulcer	To test previously designed Temperature and Pressure Monitoring and Regulating Insoles (TAPMARI) in diabetic neuropathic and healthy subjects.	8 individuals (5 healthy and 3 with DN)	28.7 (Healthy control) + 71.2 (Diabetic Neuropathy)	4 hours + 5 minutes	The results revealed that in diabetic patients, TAPMARI provided a 4°C temperature relief between the contralateral feet.
Zhu et al. (1993), United States	Diabetic foot ulcer	To quantitatively examine and compare in-shoe plantar pressures during continuous walking by normal sensate and diabetic insensate subjects.	20 sensate and 5 diabetic insensate subjects were studied	30.4 (sensate) + 67.8 years (diabetic insensate)	Variable	From the study of the coefficients of variation, the authors demonstrated a larger step-to-step variation in plantar pressures for the insensate during continuous walking, suggesting the need for caution in interpreting the data from isolated force plate steps when studying insensate individuals.
